# Compliance with antibiotic prophylaxis guidelines in a university hospital’s neurosurgical unit: a 7-year follow-up

**DOI:** 10.1186/2047-2994-4-S1-P80

**Published:** 2015-06-16

**Authors:** A Agostinho, F Olearo, D Delannoy, F Fouchard-Chavatte, M-C Goascoz, M Ares, I Cabrilo, O Gautschi, E Tessitore, P Bijlenga, C Czarnetzki, C Lysakowski, K Schaller, S Harbarth

**Affiliations:** 1 Infection Control Program, Hôpitaux Universitaires de Genève (HUG), Genève 14, Switzerland; 2 Neurosciences Department, Hôpitaux Universitaires de Genève (HUG), Genève 14, Switzerland; 3 Operating Room Management, Hôpitaux Universitaires de Genève (HUG), Genève 14, Switzerland; 4 Internal Control & Audit Service, Hôpitaux Universitaires de Genève (HUG), Genève 14, Switzerland; 5 Anesthesiology Department, Hôpitaux Universitaires de Genève (HUG), Genève 14, Switzerland

## Introduction

Surgical site infections (SSIs) are costly complications in neurosurgical practice and can be prevented by adequate perioperative antibiotic administration. Antibiotic prophylaxis (ABP) guidelines for neurosurgical procedures have been in place for 10 years at the HUG nevertheless the long-term compliance with these guidelines has not been evaluated.

## Objectives

To assess the appropriate use and guideline-compliance of ABP in neurosurgery in 2007 versus 2014.

## Methods

We performed 2 surveys on the adequacy of ABP including surveillance data on spinal and cranial surgeries collected during a 4-month period in 2007 compared with a 2^nd^ period (1.10.13 to 30.09.11) addressing spinal surgeries only. Adequate ABP was defined as correct choice and dosage of the AB (considering also MRSA carriers), optimal timing of the administration (< 1h before surgical incision) and a second dose administration of intraoperative ABP if the operation lasts > 4 hours.

## Results

In the survey of 2007, 177 operations were included compared to 314 operations in 2014. Overall, we noticed improvement in guideline compliance over the last 7 years. In 2007 ABP was omitted in 16% (28/177) of the interventions compared to 2% (7/314) in 2014. The choice and dose of the AB remained adequate over the years (98% (146/149) of the operations in 2007 versus 99% (304/307) in 2014). Improvement in the timing was also noted, passing from 52% (77/149) of the procedures with the ABP administration within the 1^st^ hour, to 81% (248/307) during the 2^nd^ period. In 2007, ABP was administrated too early (> 1 hour before the incision) in many cases. In 2014, more patients (39%, 7/18 vs 12%, 2/17) did not receive the repeated dose of ABP when required.

## Conclusion

We report an improvement of the compliance with ABP guidelines in neurosurgery in particularly regarding antibiotic timing and coverage. More efforts are needed to further optimize ABP in high-risk patients and procedures.

## Disclosure of interest

None declared.

